# Genome-wide identification and expression analysis of the *PP2C* gene family in *Apocynum venetum* and *Apocynum hendersonii*

**DOI:** 10.1186/s12870-024-05328-6

**Published:** 2024-07-09

**Authors:** Jiayi Chen, Yue Wang, Yongmei Wu, Xiaoyu Huang, Xiaojun Qiu, Jikang Chen, Qian Lin, Haohan Zhao, Fengming Chen, Gang Gao

**Affiliations:** 1 Hunan Provincial Key Laboratory of the Traditional Chinese Medicine Agricultural Biogenomics, Changsha Medical University, Changsha, 410219 China; 2 Institute of Bast Fiber Crops, Chinese Academy of Agricultural Sciences, Changsha, 410205 China; 3 Yuelushan Laboratory, Changsha, 410082 P.R. China

**Keywords:** *Apocynum*, Protein phosphatase 2 C, Salt stress

## Abstract

**Background:**

Protein phosphatase class 2 C (PP2C) is the largest protein phosphatase family in plants. Members of the *PP2C* gene family are involved in a variety of physiological pathways in plants, including the abscisic acid signalling pathway, the regulation of plant growth and development, etc., and are capable of responding to a wide range of biotic and abiotic stresses, and play an important role in plant growth, development, and response to stress. *Apocynum* is a perennial persistent herb, divided into *Apocynum venetum* and *Apocynum hendersonii*. It mainly grows in saline soil, deserts and other harsh environments, and is widely used in saline soil improvement, ecological restoration, textiles and medicine. *A. hendersonii* was found to be more tolerant to adverse conditions. The main purpose of this study was to investigate the *PP2C* gene family and its expression pattern under salt stress and to identify important candidate genes related to salt tolerance.

**Results:**

In this study, 68 *AvPP2C* genes and 68 *AhPP2C* genes were identified from the genomes of *A. venetum* and *A. hendersonii*, respectively. They were classified into 13 subgroups based on their phylogenetic relationships and were further analyzed for their subcellular locations, gene structures, conserved structural domains, and cis-acting elements. The results of qRT-PCR analyses of seven *AvPP2C* genes and seven *AhPP2C* genes proved that they differed significantly in gene expression under salt stress. It has been observed that the *PP2C* genes in *A. venetum* and *A. hendersonii* exhibit different expression patterns. Specifically, *AvPP2C2, 6, 24, 27, 41* and *AhPP2C2, 6, 24, 27, 42* have shown significant differences in expression under salt stress. This indicates that these genes may play a crucial role in the salt tolerance mechanism of *A. venetum* and *A. hendersonii*.

**Conclusions:**

In this study, we conducted a genome-wide analysis of the *AvPP2C* and *AhPP2C* gene families in *Apocynum*, which provided a reference for further understanding the functional characteristics of these genes.

**Supplementary Information:**

The online version contains supplementary material available at 10.1186/s12870-024-05328-6.

## Background

Plants are exposed to different environmental adversities such as drought, salinity, and high temperature during growth and development, which can affect the balance of ions, regulation of hormones, and the reception and transmission of signals in the plant body [1, 2]. These factors ultimately impact the growth and development of the plant and can significantly reduce crop yield and quality [3–6]. Plants have evolved an array of response mechanisms to thrive in challenging environments. Among these is protein reversible phosphorylation, a complex process that entails two opposing chemical reactions: protein phosphorylation and dephosphorylation. This modification process is facilitated by protein kinases (PKs) and protein phosphatases (PPs), and can promptly adjust the biological properties of proteins based on external stimuli. It plays a crucial role in plant growth and development, signal transduction, and in mitigating the effects of stress due to unfavorable conditions [7]. Phosphorylation occurs at specific sites in proteins, including serine (Ser), threonine (Thr), and tyrosine (Tyr), and is regulated by protein kinases (PKs) and protein phosphatases (PPs). PPs are classified based on their substrate specificity, with Ser/Thr phosphatases (STPs), protein Tyr phosphatases (PTPs), and dual-specificity phosphatases (DSPTPs) being the main categories [8]. PTPs are further divided into two groups, phosphoprotein metal phosphatases (PPM) and phosphoprotein phosphatases (PPP), based on crystal structure, amino acid sequence, and response to inhibitors [9, 10]. The PPP family includes various types of protein phosphatases, such as PP1, PP2A, PP2B, PP4, PP5, PP6, and PP7, while type 2 C protein phosphatase (PP2C) is the largest protein phosphatase family in plants. Although closely related to the PPP family, PP2C has no sequence homology and belongs to the PPM family [9–12]. PP2C has a conserved catalytic structure at the C-terminus and a functional extension region at the N-terminal that determines its various functions [12]. In *Arabidopsis*, distinct subgroups of *PP2C* genes exhibit specific functions. It is worth noting that these genes belong to the *PP2C* family, which plays a vital role in regulating plant growth and development. Group A comprises genes that participate in abscisic acid (ABA) signaling, such as *ABI1* and *ABI2*. These genes interact with *SnRK2*, binding and dephosphorylating it, which inhibits the ABA signaling pathway and stress response [13–16]. Group B is responsible for mitogen-activated protein kinase (MAPK) signaling, while Group C is mainly involved in flower development [17]. Members of Group D may have negative effects on cell elongation, but can also have positive effects on the ABA signaling pathway in response to saline stress [18–20]. Group E is responsible for regulating the opening and closing of plant stomata, while Group F induces stress responses in bacteria. The *PP2C* gene family has been extensively researched in various plants, such as rice [21, 22], maize [23, 24], and wheat [25], among others. Their roles in plant growth, development, and ability to combat negative stressors are increasingly being comprehended.

*Apocynum* is a perennial, persistent herb that grows mainly in harsh environments such as saline soils and deserts, and is widely used in saline land improvement, ecological restoration, textile and medicine [26–32]. *Apocynum* is divided into *Apocynum venetum* and *Apocynum hendersonii*, and these two species differ in morphological characteristics and physiological properties. The former is characterized by red stems and flowers, while the latter is more salt tolerant and sports white flowers, making it an ideal choice for soil improvement and restoration [33, 34]. Currently, a comprehensive systematic analysis of the PP2C gene family in *A. venetum* and *A. hendersonii* remains elusive, particularly with regards to their expression patterns and underlying functional mechanisms in response to salt stress. Therefore, studying the role and mechanism of the *PP2C* gene family in *Apocynum* under salt stress is of significant importance for improving the salt tolerance of plants.

In this study, we performed genome-wide identification and analysis of *PP2C* gene family members in *Apocynum*. 68 *AvPP2C* genes and 68 *AhPP2C* genes were identified, and the physicochemical properties, gene structure, conserved structural domains, cis-acting elements, chromosomal location distribution, gene duplication, and phylogenetic relationships of *PP2C* gene family members were analyzed. And the expression patterns of *PP2C* genes in different tissues of *Apocynum* were analyzed based on the transcriptome data of our group. The response of the *PP2C* gene after NaCl stress was analyzed by qRT-PCR study. The results of this study laid the foundation for the study of *PP2C* gene function in *Apocynum* and provided a reference for the excavation and genetic improvement of *Apocynum* resistance genes.

## Results

### Identification, characteristics and phylogenetic analysis of the *PP2C* gene family in *Apocynum*

**Fig. 1:**
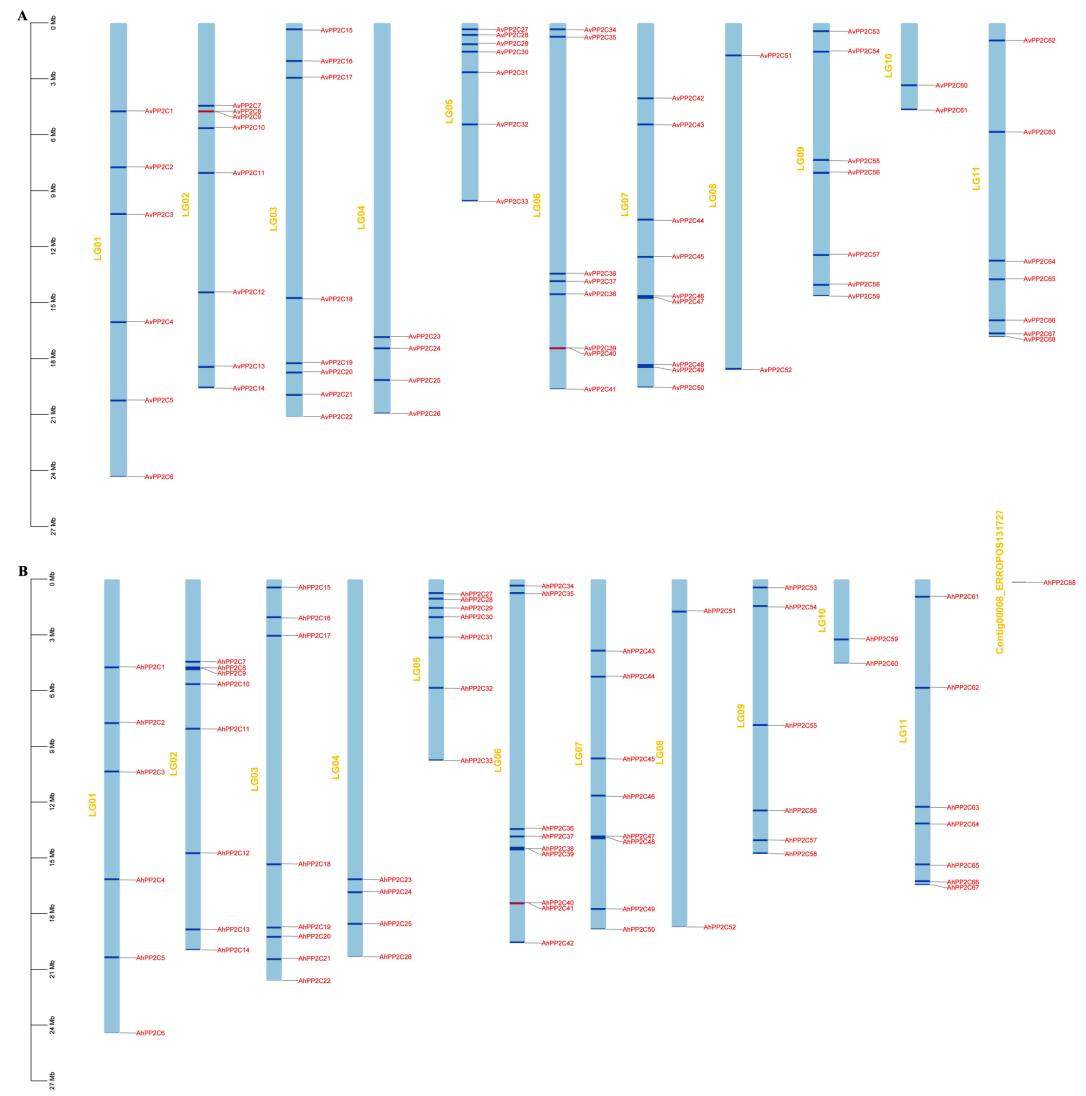
Chromosomal distribution and localization of the *PP2C* gene family in *A. venetum* and *A. hendersonii*. The blue rectangular bars represent chromosomes of *Apocynum*, and chromosome names are shown on the left side of each chromosome. The 0–27 Mb scale bar on the left represents chromosome length. (**A**) Chromosome distribution and localization of *AvPP2Cs*. (**B**) Chromosome distribution and localization of *AhPP2Cs*

In this study, we used BLASTp to identify 68 members of the *AvPP2C* gene family in *A. venetum* genome and 68 members of the *AhPP2C* gene family in the *A. hendersonii* genome, respectively, using the sequences of 80 AtPP2C proteins from *Arabidopsis thaliana* as a reference. We used TBtools to map the positional distribution of the *PP2C* genes on chromosomes of *A. venetum* and *A. hendersonii* respectively, which were named *AvPP2C1-AvPP2C68* and *AhPP2C1-AhPP2C68* according to their chromosomal positions (Fig. 1). Except for *AhPP2C68* which was not assembled, the remaining 68 *AvPP2C* genes and 67 *AhPP2C* genes were located on 11 chromosomes, respectively. There were fewer genes on chromosomes 4, 8 and 10.

We analyzed the physicochemical properties such as molecular weight (MW), theoretical isoelectric point (pI), and subcellular localization of the 68 AvPP2C proteins and 68 AhPP2C proteins (Table S1). The 68 *AvPP2C* genes encoded proteins were located at lengths ranging from 278 to 1086, theoretical isoelectric points ranging from 4.38 to 9.78, and molecular weights ranging from 30369.58 to 123024.02 Da. The 68 *AhPP2C* genes encoded proteins with lengths ranging from 138 to 1092, theoretical isoelectric points ranging from 4.62 to 9.8, and molecular weights of 15540.2-123582.74 Da. The results of subcellular localization show that *PP2C* genes have the ability to appear in various areas of the cell, including the nucleus, chloroplast, cell wall, cell membrane, cytoplasm, mitochondria, and peroxisome. Some specific *PP2C* genes, such as *AvPP2C9, 10, 16, 19, 20, 21, 29, 31, 35, 38, 47, 53, 62* and *AhPP2C9, 10, 21, 29, 31, 35, 37, 38, 39, 48, 53, 54, 58* are predicted to be present in multiple cellular structures.

**Fig. 2:**
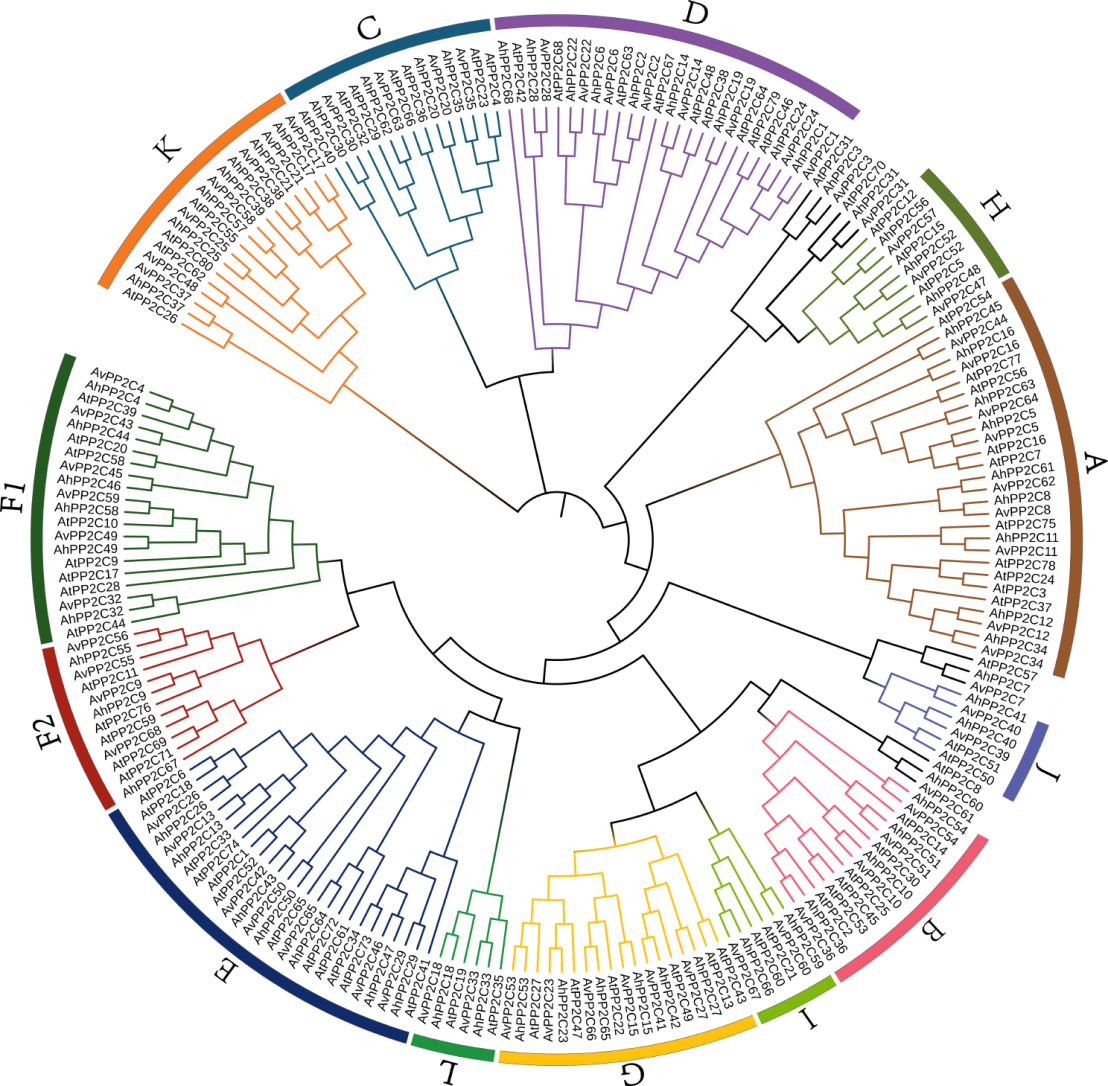
Phylogenetic analysis of *PP2C* family between *A. venetum*, *A. hendersonii* with *Arabidopsis*. The phylogenetic tree was constructed by MEGA 7 using the neighbor-joining method. The subgroups (**A**-**L**) are represented by different colors

To investigate the phylogenetic relationship between *A. venetum*, *A. hendersonii* and *Arabidopsis thaliana PP2C* genes, a phylogenetic tree was constructed using the neighbor-joining (NJ) method based on 80 *AtPP2C* genes of *Arabidopsis thaliana*, 68 *AvPP2C* genes of *A. venetum* and 68 *AhPP2C* genes of *A. hendersonii* (Fig. 2). Based on phylogenetic analysis, the *PP2C* genes in *Apocynum* were categorized into 13 subgroups (A-L). These subgroups contained 9, 4, 4, 8, 7, 6, 4, 6, 3, 2, 2, 7, 2 *AvPP2C* genes and 9, 4, 4, 9, 7, 6, 3, 6, 3, 2, 2, 7, 2 *AhPP2C* genes, with similar groupings to those found in *Arabidopsis*. Notably, PP2C proteins from both *Apocynum* and *Arabidopsis* were present in each subfamily, suggesting a common ancestor. Interestingly, *AvPP2C3*, *AvPP2C7*, *AvPP2C31*, *AvPP2C61*, *AhPP2C3*, *AhPP2C7*, *AhPP2C31*, and *AhPP2C60* of *Apocynum* did not cluster with any other group. The evolutionary tree also showed that genes in *A. venetum* and *A. hendersonii* were tightly clustered, indicating high homology and similar gene functions.

### Gene structures and conserved motifs analyses of the *AvPP2C* genes and the *AhPP2C* genes

**Fig. 3:**
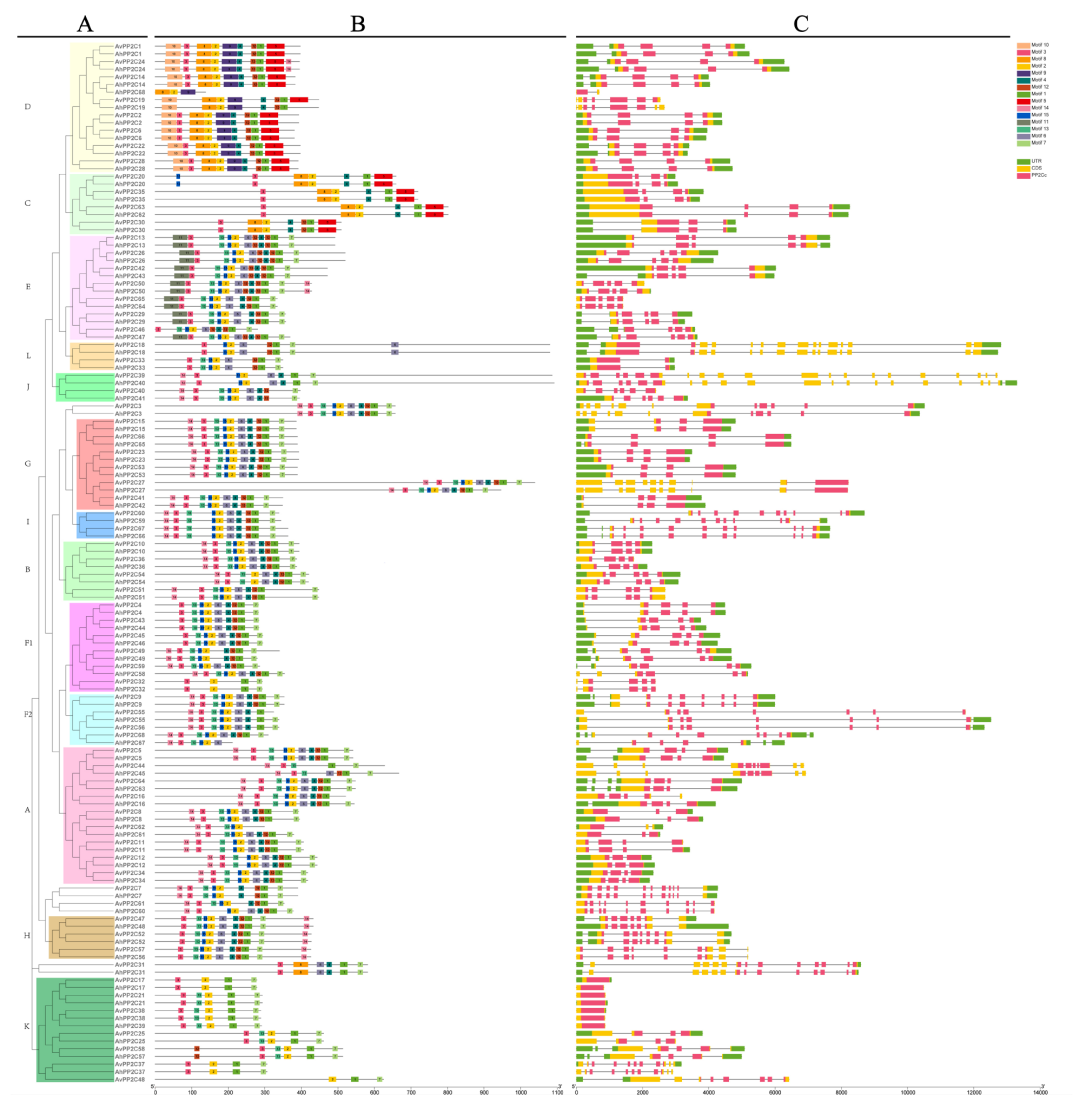
Phylogenetic tree, conserved domains and gene structures of 68 AvPP2Cs proteins and 68 AhPP2Cs proteins. (**A**) The phylogenetic tree was constructed using all AvPP2C proteins and AhPP2C proteins with 1000 replicates on each node. (**B**) The conserved domains identified by the Batch CD-search Tool of the NCBI website. 15 colored squares represent motif1-motif15 and gray lines represent amino acid lengths. (**C**) The gene structures of *AvPP2C* genes and *AhPP2C* genes. The green rectangles are UTRs, yellow rectangles are CDSs, and pink rectangles are *PP2C* structural domains, and the gray lines represent introns

Based on their phylogenetic relationships, the structures and conserved domains of the *AvPP2C* and *AhPP2C* genes were studied. The gene structures include positional information such as coding sequence (CDS), untranslated regions (UTR), introns and *PP2C* structural domains (Fig. 3A and C). Genes within the same subgroup share similar gene structures, but there are variations in the length and arrangement of their CDSs and UTRs. For instance, all *PP2C* genes in group C contained UTR and CDS regions, but their lengths varied. *AvPP2C42* had the lengthiest UTR region, while 16 genes in *A. venetum* had no UTR regions and 14 genes in *A. hendersonii* had no UTR regions. *AvPP2C39* and *AhPP2C40* had the highest number of CDS regions, totaling 16. After examining the number of introns, it was found that three *AvPP2C* genes and four *AhPP2C* genes in *Apocynum* had no introns, while the remaining 129 *PP2C* genes contained introns.

In order to study the structure and function of PP2C proteins and their evolutionary relationships, the conserved domains of AvPP2Cs proteins and AhPP2Cs proteins were analyzed using the online MEME website. The analysis revealed 15 different conserved domains, which were given the names motif 1 to motif 15 (Fig. 3A and B, Table S2). The results demonstrated that PP2C proteins in the same subgroups possess a similar distribution that may be linked to their functions. Motif 1 (except AvPP2C62, AhPP2C67, AhPP2C68), motif 2 (except AvPP2C31, AvPP2C44, AhPP2C45, AhPP2C31), and motif 3 (except AhPP2C68, AhPP2C19, AvPP2C19, AvPP2C48) were present in all subgroups. Motifs 6, 7, 11, 13 and 15 were absent in subgroups C and D, while motif 5 was only present in subgroups C and D, except AhPP2C68. Motif 9 and 10 were present only in subgroup D, and motif 11 was present only in subgroup E, but not in other groups.

### Cis-element analysis of the *PP2Cs* promoter in *A. venetum* and *A. hendersonii*

**Fig. 4:**
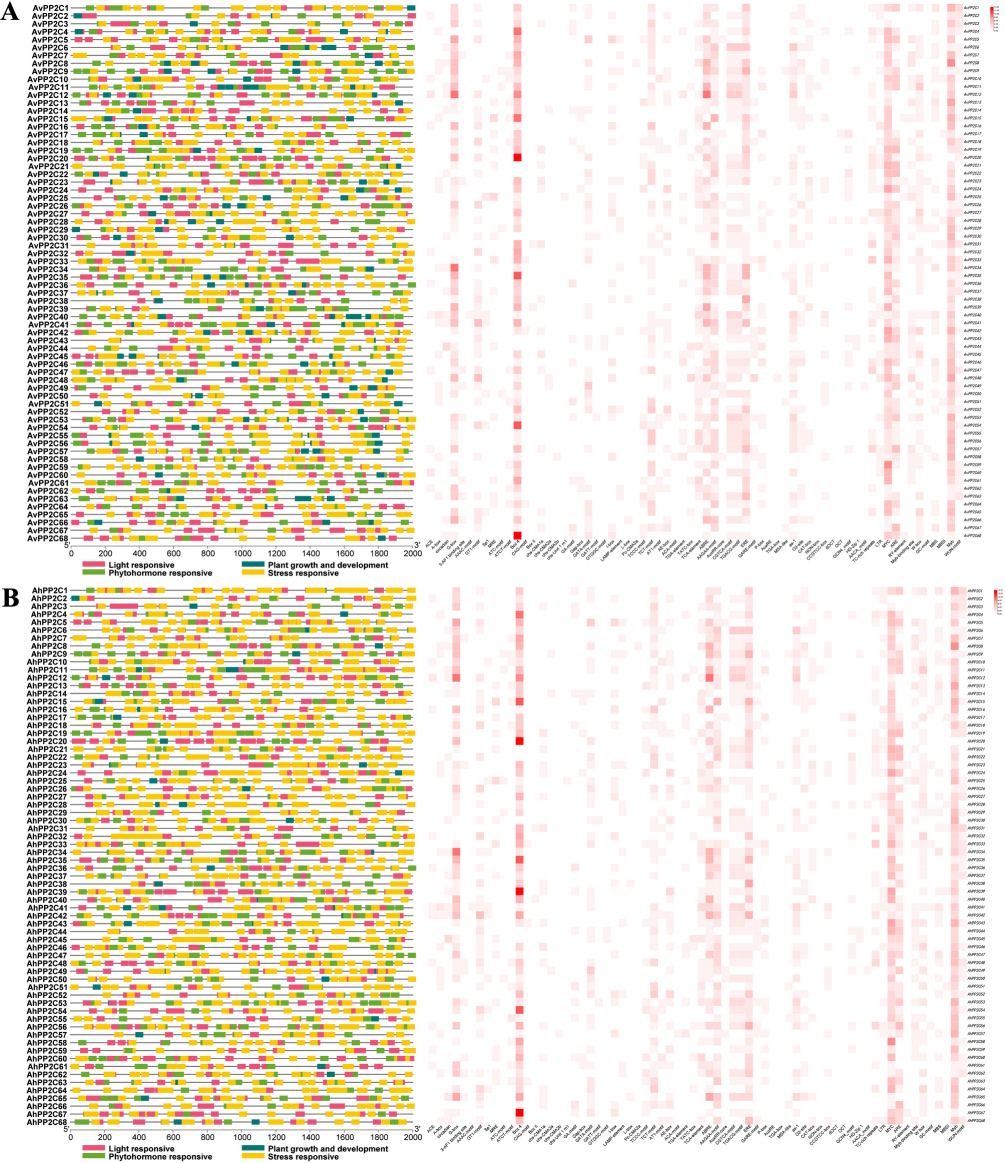
Analysis of cis-acting elements in the promoter region of *AvPP2Cs* and *AhPP2Cs*. The distribution of cis-acting elements in the promoter region is shown on the left. On the right side is the heat map of cis-acting elements for the light-responsive, phytohormone-responsive, and-responsive, and the color concentration of the squares indicates the number of cis-acting elements. (**A**) Analysis of cis-acting elements in the promoter region of *AvPP2Cs*. (**B**) Analysis of cis-acting elements in the promoter region of *AhPP2Cs*

Using the PlantCARE online database, we analyzed and predicted the cis-acting elements in the promoter regions of *A. venetum* and *A. hendersonii*, and conducted a visual analysis using TBtools software. A total of 68 cis-acting elements were retrieved. Among them, there were 32 light-responsive elements, 13 phytohormone-responsive elements, 12 stress-responsive elements, and 11 elements related to plant growth and development (Fig. 4, Table S3). It was found that all the promoters of *AvPP2Cs* and *AhPP2Cs* contained light-responsive elements, phytohormone-responsive elements, and stress-responsive elements. However, elements associated with plant growth and development were missing in *AvPP2C18*, *AvPP2C33*, *AvPP2C59*, *AvPP2C61*, *AvPP2C65*, *AhPP2C18*, *AhPP2C19*, *AhPP2C32*, *AhPP2C58*, *AhPP2C60*, and *AhPP2C66*. Box 4 was identified as the most abundant light-responsive element, with 263 in *A. venetum* and 290 in *A. hendersonii*. This suggests that the expression of *PP2C* genes may be strongly influenced by light conditions. Among the phytohormone response elements, a large number of abscisic acid response elements (ABRE) and AAGAA-motifs were found, with *AvPP2Cs* having 148 ABREs and 132 AAGAA-motifs, and *AhPP2Cs* having 141 ABREs and 129 AAGAA-motifs. The presence of these elements suggests that *PP2C* genes play an important role in ABA-mediated stress resistance pathways. The ethylene response element (ERE) was also found in large numbers, with *AvPP2Cs* having 147 and *AhPP2Cs* having 156. This indicates that the ethylene signaling pathway may be involved in the regulation of *PP2C* gene expression. The stress-responsive elements MYB and MYC were found to be the most abundant with 247 and 235 in *A. venetum* and 242 and 238 in *A. hendersonii*, respectively. These elements are involved in drought and ABA-induced responses. Another stress-responsive element, ARE, was found to be essential for anaerobic induction, with 133 in *A. venetum* and 130 in *A. hendersonii*. The large number of these elements suggests that most *PP2C* genes may respond to various stresses and are closely related to plant stress resistance. In *A. venetum* and *A. hendersonii*, the number of the same type of cis-acting elements differs, reflecting subtle differences in gene expression regulation between the two plants, which may be related to their ecological adaptability or genetic background.

### Collinear analysis and evolutionary analysis of *AvPP2Cs* proteins and *AhPP2Cs* proteins

**Fig. 5:**
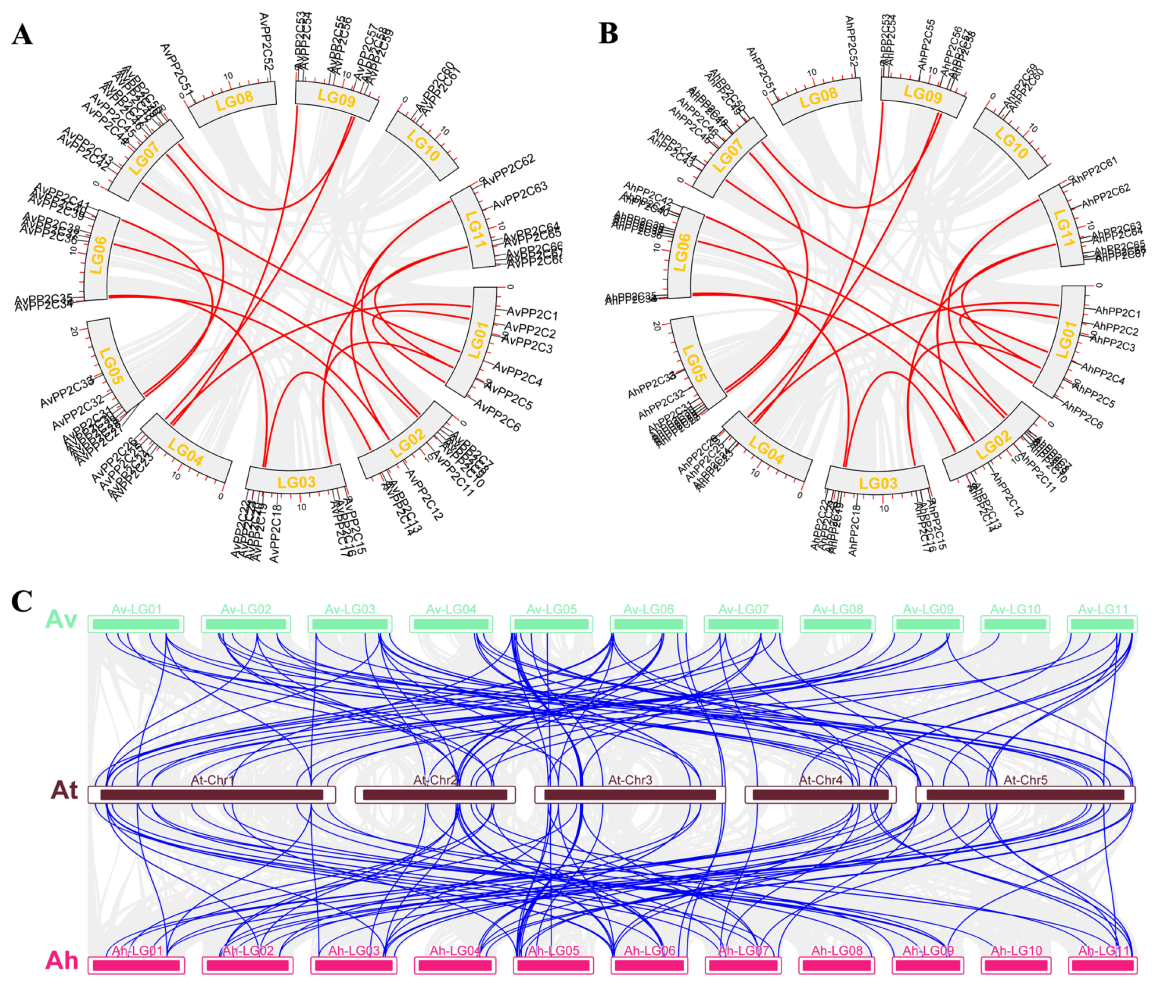
Collinearity analysis of the *AvPP2C* genes. (**A**) Distribution of *AvPP2C* genes on chromosomes and gene duplications. Red lines represent gene fragment duplications. (**B**) Distribution of *AhPP2C* genes on chromosomes and gene duplications. Red lines represent gene fragment duplications. (**C**) Comparative analysis of *PP2C* genes between *Arabidopsis* and *A. hendersonii* and *A. venetum*. Blue lines represent homologs of *PP2C* genes

The expansion of gene families in the genome is predominantly caused by tandem and segmental duplications. To gain insight into the *PP2C* gene family, 68 *AvPP2C* genes and 68 *AhPP2C* genes were analyzed for duplication (Fig. 5A and B, Table S4). It was discovered that in *A. venetum*, one tandem duplication pair was located on chromosome LG06 (39/40), whereas 17 pairs of segmental duplication genes were unevenly distributed on chromosomes other than LG08 and LG10. In *A. hendersonii*, there were 2 tandem replication gene pairs located on chromosome LG06 (38/39, 40/41), and 17 fragment replication gene pairs were unevenly distributed on 9 chromosomes except for LG08 and LG10. These findings suggest that the evolution of *PP2Cs* in *Apocynum* was primarily driven by segmental duplication events, as the number of segmental duplication gene pairs was significantly greater than that of tandem duplications.

Furthermore, the study investigated the homology between *A. venetum*, *A. hendersonii*, and *Arabidopsis thaliana* to understand the evolutionary relationship of *PP2C* genes (Fig. 5C, Table S5). The results indicated that there were 84 homologous gene pairs between *A. venetum* and *Arabidopsis thaliana*, and 82 homologous gene pairs between *A. hendersonii* and *Arabidopsis thaliana*. Among these pairs, *A. venetum* and *A. hendersonii* had the highest number of homologous gene pairs on chromosome LG06, with 13 pairs each. These findings suggest that *A. venetum* and *A. hendersonii* may have a high degree of homology with *Arabidopsis*.

### Tissue-specific expression of *AvPP2C* genes and *AhPP2C* genes in different tissues

**Fig. 6:**
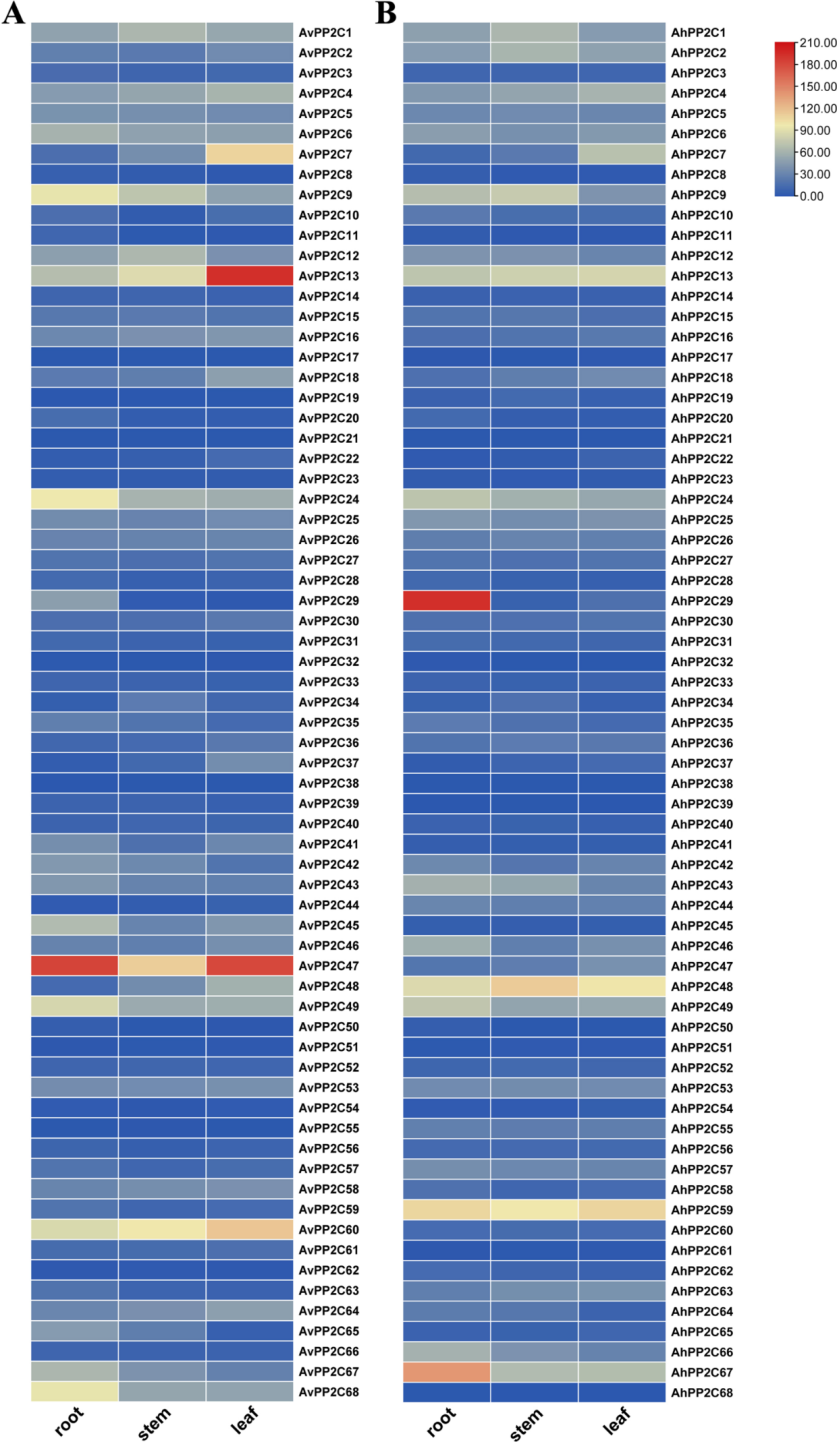
Expression patterns of *AvPP2C* genes (A) and *AhPP2C* genes (B) in different tissues (root, stem and leaf). Heatmaps of *AvPP2Czs* and *AhPP2Cs* RNA-seq data in three tissues were plotted using TBtools, with red and blue boxes representing high and low levels of expression, respectively

To gain a better understanding of the function of *PP2C* genes in the growth and development of *A. venetum* and *A. hendersonii*, we analyzed the expression patterns of *PP2C* genes using RNA-Seq data of roots, stems, and leaves of both species (Fig. 6). Most of the *PP2C* genes were expressed in all tissues, except for four genes (*AvPP2C19*, *AvPP2C38*, *AhPP2C39*, and *AhPP2C68*), which were barely expressed. In *A. venetum*, *AvPP2C47* was highly expressed in roots, stems, and leaves. *AvPP2C9*, *AvPP2C24*, *AvPP2C49*, *AvPP2C60*, and *AvPP2C68* were moderately expressed in roots; *AvPP2C13* and *AvPP2C60* were highly expressed in stems; *AvPP2C13* was highly expressed in leaves, and *AvPP2C7* and *AvPP2C60* were moderately expressed in leaves. In *A. hendersonii*, *AhPP2C29* and *AhPP2C67* were highly expressed in roots, while *AhPP2C24*, *AhPP2C13*, *AhPP2C49*, *AhPP2C48*, and *AhPP2C59* were moderately expressed in roots. *AhPP2C9*, *AhPP2C13*, *AhPP2C59*, and *AhPP2C48* were highly expressed in stems, and *AhPP2C13*, *AhPP2C48*, and *AhPP2C59* were highly expressed in leaves.

### Relative expression changes of *AvPP2C* genes and *AhPP2C* genes under NaCl treatment

**Fig. 7:**
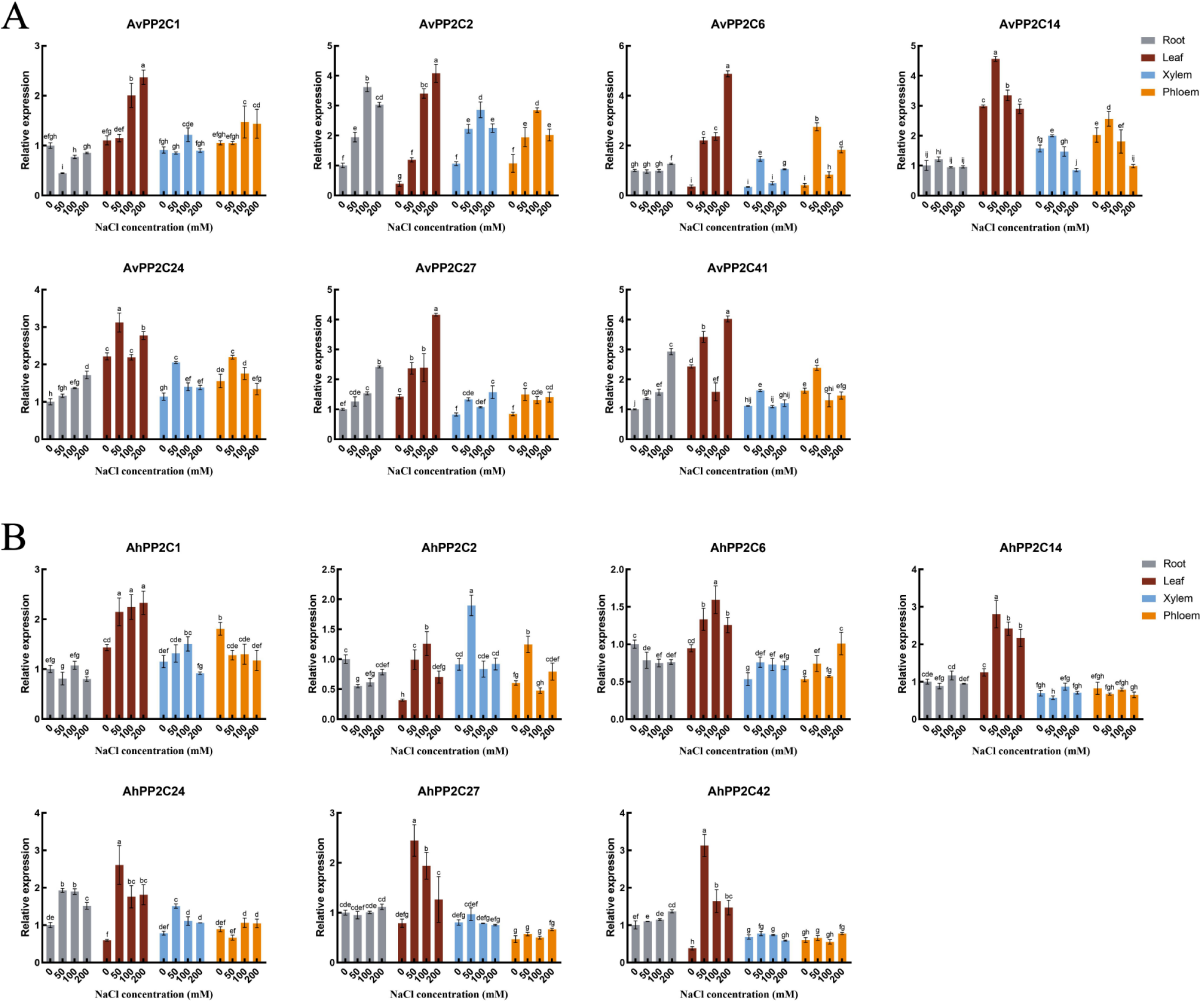
The qRT-PCR results of selected *PP2C* genes of different tissue samples (root, xylem, phloem, leaf) of *A. venetum* and *A. hendersonii* after 14 days of NaCl stress. (**A**) The relative expression levels of selected *AvPP2C* genes in *A. venetum* parts. (**B**) The relative expression levels of selected *AhPP2C* genes of *A. hendersonii*

In order to study the expression of *AvPP2C* genes and *AhPP2C* genes under salt stress conditions and to identify important *PP2C* genes related to salt tolerance. Based on phylogenetic analysis and homology of known *PP2C* genes in *Arabidopsis thaliana*, five genes in subgroup D and two genes in subgroup G were selected in *A. hendersonii* and their expression patterns in different tissues of *A. venetum* under salt stress were investigated by qRT-PCR (Fig. 7).

The results showed that the *AvPP2C1* gene was down-regulated in roots after NaCl stress, with the lowest expression at 50 mM concentration. It was up-regulated in leaves and phloem, with the highest expression at 200 mM and 100 mM concentrations, respectively, reaching 2.14-fold and 1.40-fold of CK. *AhPP2C1* gene was up-regulated in leaves and increased with increasing NaCl concentration; it showed down-regulation in bast and decreased with increasing NaCl concentration. *AvPP2C2* gene showed up-regulation under NaCl stress, in which the expression of the gene in leaves varied the most with NaCl concentration, which was 10.44 times that of CK. The expression trend of *AvPP2C2* in root, xylem and phloem was the same, and all of them reached the maximum expression at 100 mM concentration. While the *AhPP2C2* gene was down-regulated in roots and up-regulated in leaves after NaCl stress, there was little change in the xylem and phloem, but the expression reached the maximum at 50 mM concentration in all. *AvPP2C6* gene did not change significantly in roots, but showed up-regulation in leaves and xylem phloem, and it is noteworthy that its expression in leaves increased with the increase of NaCl concentration and reached the maximum at 200 mM, which was 13.55 times that of CK. And *AhPP2C6* was different from *AvPP2C6* in that it showed down-regulation in roots. The expression of *AvPP2C14* was different in roots, leaves, xylem and phloem, with the highest in leaves and the lowest in roots; after NaCl stress, the *AvPP2C14* gene showed a tendency of increasing and then de-creasing with the increase of NaCl concentration, and its expression reached the maximum in all tissues at the concentration of 50 mM. While the expression of *AhPP2C14* did not change significantly under NaCl treatment, it only showed up-regulation in leaves, and the expression of the gene was the highest at 50 mM concentration, and its expression gradually declined with the increase of concentration. *AvPP2C24* gene had the highest expression in leaves and showed up-regulation in all tissues after NaCl stress. *AhPP2C24* gene was the same as *AvPP2C24* gene in that the expression of *AhPP2C24* gene was up-regulated in all tissues after NaCl stress, except in phloem at 50 mM concentration. The difference was that the expression of *AhPP2C24* gene changed more significantly in leaves after NaCl treatment. Both *AvPP2C27* and *AhPP2C27* genes showed up-regulation after NaCl treatment, but the expression of *AvPP2C27* gene in roots and leaves increased with increasing NaCl concentration, while the expression of *AhPP2C27* gene in leaves reached a maximum at 50 mM concentration and then decreased with increasing concentration. The expression of *AvPP2C41* and *AhPP2C42* genes in roots increased with increasing NaCl concentration, *AvPP2C41* gene showed down-regulation in leaves at 100 mM and up-regulation at 50 mM and 200 mM concentrations. While *AhPP2C42* gene showed up-regulation after NaCl stress, the expression was maximum in leaves at 50 mM concentration, which was 8.20 times of CK, and then decreased with the increase of NaCl concentration.

## Discussion

*PP2C* genes are closely related to abscisic acid ABA signaling pathway, plant adversity stress, and stomatal opening and closing, etc. The *PP2C* gene families of *Arabidopsis thaliana*, rice, maize, and wheat have been investigated by previous authors. In this study, we comprehensively analyzed the *AvPP2C* genes of *A. venetum* and the *AhPP2C* genes of *A. hendersonii*, including the identification of the gene families, phylogenetic relationships, and chromosomal locations, collinear relationship, gene structure, conserved motifs, and expression patterns. A total of 136 *PP2C* genes, including 68 *AvPP2C* genes and 68 *AhPP2C* genes, were identified in *Apocynum*, and the physicochemical properties and subcellular localization of the genes were predicted, and it was found that the vast majority of *PP2C* genes were located in the nucleus and chloroplasts, and a few genes might be present in the mitochondria, cell wall, cytoplasm, peroxisomes, and cell membrane. Therefore, it is hypothesized that they may be related to photosynthesis, cell growth and development, and respiration. According to phylogenetic and evolutionary relationship analysis, *A. hendersonii* and *A. venetum* are closely related to each other, and a total of 136 *PP2C* genes in *A. hendersonii* and *A. venetum* were divided into 13 subgroups (A-L), which is consistent with the grouping of *Arabidopsis thaliana*. Previous studies found that genes in the A subgroup of *PP2C* genes in *Arabidopsis* mainly inhibit ABA receptor activity and negatively regulate ABA signaling [35]; the B subgroup is involved in the mitogen-activated protein kinase signaling pathway and participates in the process of MAPK phosphorylation, and is capable of dephosphorylating and inactivating MAPK [36]; Subgroup C gene family members are involved in stem cell maintenance and differentiation [37]; Members of subgroup D negatively regulate small auxin up RNA (SAUR)-mediated cell elongation, and PP2C.D1, PP2C.D2, and PP2C.D5 in Arabidopsis act during leaf greening, and PP2C.D1 regulates hook formation by affecting ethylene accumulation [18, 38, 39]. Moreover, members of this group respond to saline and alkaline stress, which may be related to stomatal movement and function, by inhibiting plasma membrane H-ATPase activity. It has been shown that *Arabidopsis* PP2C.D negatively regulates aluminium resistance by modulating malate secretion [19, 40]; Members of the E subgroup gene family may be associated with inflorescence stem growth, regulation of stomatal signaling, and so on [41–43]. Based on the phylogenetic relationship between *Apocynum* and *Arabidopsis*, it is hypothesized that the functions of gene family members located in the same subgroup in *Apocynum* may be similar to those in *Arabidopsis*. The prediction results of subcellular localization for *PP2C* gene family members in *Apocynum* indicate that most *PP2C* genes in *Apocynum* are located in the nucleus and chloroplasts. This suggests that *PP2C* genes may participate in regulating the expression of salt stress-related genes in the nucleus, and may also be involved in regulating the activity of enzymes related to photosynthesis. Additionally, some genes may be located in the cell wall, cytoplasm, cell membrane, mitochondria, and peroxisomes, which may be closely related to reducing water loss, regulating intracellular ion concentration, and scavenging reactive oxygen species in plant cells. These functions can enhance the salt tolerance of plants. The predictions of *PP2C* gene family in *A. venetum* and *A. hendersonii* were partially different. For example, *AvPP2C9* was predicted to be located in mitochondria and the nucleus, whereas *AhPP2C9* was predicted to be located in chloroplasts, mitochondria, and the nucleus. *AvPP2C20* was located in the nucleus and chloroplasts, whereas *AhPP2C20* was predicted to be located in chloroplasts only. This may imply differences in the location and function of some *PP2C* genes in *A. hendersonii* and *A. venetum*.

The diversity of gene structures, conserved structural domains, and cis-acting elements is also closely related to the functions of gene families. Genes in the same subgroup are similar in structure, which may imply that they have the same function [44]. The gene structures of *A. hendersonii* and *A. venetum* genes from the same evolutionary tree branching were similar, except for the *AhPP2C68* gene. However, the gene structures of individual *PP2C* genes were different again, for example, *AvPP2C18* and *AhPP2C18* in subgroup L had significantly more CDS regions compared with *AvPP2C33* and *AhPP2C33*, which might be related to the increase of introns during the evolutionary process. The *PP2C* gene family is evolutionarily conserved, and most *PP2Cs* have a highly conserved structural domain with a unique non-catalytic N-terminal extension region. The specificity of the function of each sub-group of *PP2C* genes may be related to an N-terminal substrate docking domain [45]. A total of 15 conserved motifs were identified in the amino acid sequences of *AvPP2C* and *AhPP2C* genes, and the distribution of conserved motifs was similar for genes in the same subgroup, which may be closely related to the function of PP2C proteins. The sequence structures of some *PP2C* genes in *A. venetum* and *A. hendersonii* exhibit differences, which may lead to variations in their functions or expression regulation. Cis-acting elements are involved in the regulation of gene expression and are closely related to plant growth and development, hormone responses and responses to various stresses. Among *AvPP2C* and *AhPP2C* genes, Box4 elements were the most abundant (AvPP2Cs: 263; AhPP2Cs: 290), followed by MYB elements (AvPP2Cs: 247; AhPP2Cs: 242). MYB elements are closely related to plant abiotic stress response [46]. Among all the response elements, light responsive and stress responsive were the most numerous elements, with a high number of 1,318 stress responsive in *A. hendersonii*. This may be related to the characteristics of *A. hendersonii* high-stress tolerance.

In this study, chromosomal position analysis and covariance analysis of *PP2C* gene family of *A. venetum* and *A. hendersonii* were carried out, which is important to understand the amplification mechanism of the genes. It was found that both *AvPP2Cs* and *AhPP2Cs* were distributed on 11 chromosomes. Seventeen pairs of segmental duplications and one pair of tandem duplications were identified in *A. venetum*; 17 pairs of segmental duplications and two pairs of tandem duplications were identified in *A. hendersonii*. Gene duplication is the main driver of gene family amplification, which mainly includes segmental duplication, tandem duplication, and genomic duplication, and segmental duplication is more conducive to maintaining gene function than tandem duplication [47]. Therefore, we hypothesize that segmental duplication is the main force driving the evolution and expansion of the *PP2C* gene family, which is similar to the results found for *PP2C* in *Arabidopsis* [48], woodland and pineapple strawberry [49], and cucumber [7].

*AvPP2C* genes and *AhPP2C* genes showed specific expression in different tissues, and most of the *PP2C* genes were lowly expressed in roots, stems and leaves. However, among all the *PP2C* genes, *AvPP2C13*, *AvPP2C47*, *AvPP2C60*, *AhPP2C48*, and *AhPP2C59* showed higher expression in all three tissues: roots, stems, and leaves, whereas *AhPP2C29*, and *AhPP2C67* showed high expression in roots and lower expression in stems and leaves. In cucumber, most of the *CsPP2C* genes were highly expressed in fertilized ovaries, male, female, and leaf (except *CsPP2C11, 41, 5, 33, 50*), and low in other tissues [4]. In maize, most of the *ZmPP2C* genes were expressed in all tissues, with *ZmPP2C42* and *ZmPP2C47* being the most highly expressed in mature pollen, and *ZmPP2C59* being expressed in primary roots and root cortex [50]. This suggests that *PP2C* genes in different plants may play different roles at different stages of plant growth, and some of these *PP2C* genes may have special functions in specific tissues.

Studies on *Arabidopsis* have demonstrated that members of subgroup D respond to saline stress, and *AtPP2C.D6* and *AtPP2C.D7* interact with *SCaBP8* of the SOS pathway, whose phosphatase activity is inhibited under salt stress. *AtPP2C.G1* in group G is dependent on ABA to positively regulate salt tolerance. Our study revealed that the expression of certain *AvPP2C* genes differed significantly from that of *AhPP2C* genes following NaCl stress. Specifically, the expression of the *AvPP2C2* gene was significantly up-regulated after salt stress, and its expression was notably higher in roots, xylem, phloem, and leaves. It indicated that *AvPP2C2* gene might play a crucial role in these four tissues when *A. venetum* resistes salt stress. The expression of *AhPP2C2* gene was down-regulated in roots but up-regulated in leaves after salt stress.In contrast, it exhibited a similar expression pattern in xylem and phloem, peaking at a concentration of 50 mM NaCl. *AvPP2C2* and *AhPP2C2* displayed distinct expression patterns in *A. venetum* and *A. hendersonii* under salt stress, which we hypothesize is related to their varying salt tolerance mechanisms. The expression of *AvPP2C6* and *AhPP2C6* was up-regulated in leaves, xylem and phloem. The change in *AvPP2C6* expression was not apparent in roots, whereas *AhPP2C6* showed down-regulation in roots. Additionally, *AvPP2C24, 27* and *41* in roots displayed similar trends after salt stress. In summary, *PP2C* genes located within the same subgroup exhibited diverse responses to salt stress, while there may be different expression patterns observed in *A. venetum* and *A. hendersonii*, where *AvPP2C2, 6, 24, 27, 41* and *AhPP2C2, 6, 24, 27, 42* displayed significant differences in expression under salt stress. These fundings suggest that these genes may play significant roles in the salt stress response of *A. venetum* and *A. hendersonii*. Under salt stress, the significant expression changes of genes such as *AvPP2C2*, *AvPP2C6*, *AvPP2C27*, *AhPP2C27*, and *AhPP2C42* in leaves may indicate that they play diverse roles in plant salt tolerance mechanisms. These include regulating ion balance, participating in signal transduction, influencing plant growth and development, responding to stress, and activating defense mechanisms. These mechanisms work together to enhance the plant’s ability to adapt to salt stress.

*Apocynum*, renowned for its remarkable stress tolerance, holds immense potential in deciphering the role of the *PP2C* gene family under salt stress conditions. By investigating the expression patterns of these genes, we can gain insights into their specific functions in responding to salt stress. In *Apocynum*, *PP2C* genes are pivotal in stress response mechanisms, and their precise regulation through advanced gene editing techniques or molecular marker-assisted breeding approaches can facilitate the cultivation of crop varieties with enhanced stress resilience. Additionally, secondary metabolites, particularly flavonoids, in *Apocynum* exhibit significant medicinal value and play a crucial role in plant growth, development, and defense mechanisms [51]. A comprehensive understanding of the role of *PP2C* genes in the biosynthesis of these secondary metabolites could serve as a theoretical foundation for future drug research and development. In conclusion, a thorough exploration of the functional characteristics of the *PP2C* gene family in *Apocynum* offers novel insights and strategies for stress-resistant breeding, medicinal value exploitation, and other applications.

## Conclusions

In this comprehensive study, we have conducted a genome-wide identification and in-depth analysis of PP2C family members in the genomes of *A. venetum* and *A. hendersonii*. A total of 68 *AvPP2C* genes and 68 *AhPP2C* genes were successfully identified and systematically classified into 13 distinct subgroups. These subgroups were then subjected to a rigorous analysis of subcellular localization, gene structure, conserved structural domains, and cis-acting elements. Upon evaluating the expression patterns of *PP2C* genes in *A. venetum* and *A. hendersonii* under salt stress conditions, we observed significant differences, particularly in the subgroup D and subgroup G genes. These variations in expression might serve as potential indicators of the distinct salt tolerance mechanisms between *A. venetum* and *A. hendersonii*. The findings of this study offer valuable insights into the functional roles of *PP2C* genes and serve as a foundation for future research aimed at elucidating the precise mechanisms underlying salt tolerance in these plant species.

## Materials and methods

### Identification of *PP2C* genes in *Apocynum*

The protein sequences of 80 *AtPP2C* genes of *Arabidopsis* were retrieved from Tair (https://www.arabidopsis.org/) [52]. Additionally, we acquired the protein sequences of *A. venetum* and *A. hendersonii* from the whole genome data sequenced within our laboratory. To identify the *PP2C* genes, we employed two distinct methodologies. Initially, we utilized BLASTp to locate potential PP2C proteins within the genomes. Subsequently, we downloaded the PP2C structural domain (PF00481) from Pfam (http://pfam.xfam.org/) and leveraged HMMER 3.0 to search our local protein database [46, 53, 54]. Concurrently, we employed the SMART (https://smart.embl.de/) to predict the structural domain, setting E-value < e^− 5^ [55, 56]. By intersecting the results from both methods, we extracted the relevant protein sequencesfrom the local protein database using TBtools, and identified the gene IDs through rigorous screening.

### Sequence analysis and basic information of the *Apocynum PP2C* gene family

Physicochemical property analysis of PP2C proteins including number of amino acid, molecular weight, theoretical pI, instability index, aliphatic index, grand average of hydropathicity were analyzed by Protein Paramter Calc in TBtools. Prediction of the subcellular location of PP2C protein by Plant-mPloc (http://www.csbio.sjtu.edu.cn/bioinf/plant-multi/) of Cell-Ploc 2.0.

### Analysis of chromosome location and collinearity analysis

Chromosome position information was obtained from the annotation file of the *Apocynum* genome and the chromosome position distribution was mapped using TBtools. Homology and collinearity within the *A. venetum* and *A. hendersonii* genomes and between them and *Arabidopsis thaliana* were analyzed using the MCScanX software and visualized using the Advanced Circos and Multiple Synteny Plot functions in TBtools [57, 58].

### Construction of phylogenetic tree

The phylogenetic tree of the *PP2C* gene family of *Arabidopsis* and *Apocynum* was constructed using MEGA-X with the Neighbor-Joining method, Bootstrap value set to 1000 and other default parameters [59, 60]. Finally, the phylogenetic tree was embellished with iTOL(https://itol.embl.de/) [61].

### Analysis of gene structures and protein conserved motifs

The gene structure of *Apocynum PP2C* was analyzed using the Batch CD-search (https://www.ncbi.nlm.nih.gov/Structure/bwrpsb/bwrpsb.cgi) on the NCBI website. The MEME website (https://meme-suite.org/meme/tools/meme) was used to predict the conserved structural domains of the *PP2C* genes in *Apocynum*, setting the motif number to 15 and the rest to default values [62]. Gene Structure View in TBtools was used to generate the final overall map of the phylogenetic tree, gene structure and conserved structural domains of *PP2Cs*.

### Analysis of cis-acting elements in *PP2C* gene promoters

The sequences of 2.0 kb upstream of *AvPP2C* genes and *AhPP2C* genes were extracted using TBtools and then the results were submitted to PlantCARE website (https://bioinformatics.psb.ugent.be/webtools/plantcare/html/) for cis-acting element prediction [62].

### Expression analysis of *PP2C* genes

From the transcriptome data of roots, stems and leaves of *A. venetum* and *A. hendersonii*, the FPKM (Fragments Per Kilobase of exon model per Million mapped fragments) values of *AvPP2Cs* and *AhPP2Cs* were extracted and heat maps were generated using TBtools [58].

### Plant material treatment methods

Seedlings grown under hydroponic conditions for 6–8 weeks were divided into four groups and treated with different concentrations of NaCl stress (0, 50, 100, and 200 mM). After 14 days of treatment, roots, leaves, xylem and phloem samples were collected and stored in a refrigerator at -80 °C.

### RNA isolation and qRT-PCR

Total RNA was extracted using the *SteadyPure* Plant RNA Extraction Kit (Accurate Biotechnology (Changsha, China) Co., Ltd.) according to previously published protocols [63], and the RNA was reverse transcribed into cDNA using the *Evo M-MLV* One Step RT-PCR Kit (Accurate Biotechnology (Changsha, China) Co., Ltd.) according to manufacturer’s protocols [64, 65]. Specific primers were designed for qRT-PCR using Primer5 (Table S6). ACT gene was used as an internal reference gene. qRT-PCR was performed on a CFX96 Touch Deep Well Real-Time Quantitative PCR System (Bio-Rad) using SYBR^®^ Green Premix *Pro Taq* HS qPCR Kit II (Accurate Biotechnology (Changsha, China) Co., Ltd.), and performed according to published protocols [64, 66]. The relative expression of *PP2C* genes was calculated using the 2^−ΔΔCT^ method and histograms were plotted using GraphPad Prism 8.

### Electronic supplementary material

Below is the link to the electronic supplementary material.

Supplementary Material 1

Supplementary Material 2

Supplementary Material 3

Supplementary Material 4

Supplementary Material 5

Supplementary Material 6

## Data Availability

The sequence information of ArabidopsisPP2C family genes were collected from The Arabidopsis Information Resoure (https://www.arabidopsis.org/). The PP2C family expression data were generated by qRT-PCR. The datasets used and/or analyzed during the current study are available from the corresponding author on reasonable request.

## Abbreviations

PP2C: Protein phosphatase class 2 C
qRT: PCR-Quantitative Real-time PCR
MAPK: Mitogen-activated protein kinase
MW: Molecular weight
pI: Isoelectric point
CDS: Coding sequence
UTR: Untranslated regions
CK: Control check
SAUR: Small auxin up RNA
